# Non-Lethal Dose-Response Models Replace Lethal Bioassays for Predicting the Hazard of Para-Aminopropiophenone to Australian Wildlife

**DOI:** 10.3390/ani13030472

**Published:** 2023-01-29

**Authors:** Clive A. Marks, Lee Allen, Heli Lindeberg

**Affiliations:** 1 Nocturnal Wildlife Research Pty Ltd., P.O. Box 2126, Melbourne, VIC 3145, Australia; 2 Manaaki Whenua Landcare Research, Lincoln P.O. Box 69040, New Zealand; 3 Queensland Department of Agriculture Fisheries and Forestry, Toowoomba, QLD 4350, Australia; 4 Natural Resources Institute Finland (Luke), Production Systems, Halolantie 31 A, FI-71750 Maaninka, Finland

**Keywords:** para-aminopropiophenone, PAPP, methaemoglobin, MetHb, animal welfare, lethal-dose bioassay, LD_50_, non-lethal assay, replacement, 3Rs

## Abstract

**Simple Summary:**

Para-aminopropiophenone (PAPP) is registered as bait poison for the humane control of red foxes and wild dogs in Australia. To classify the relative hazard of poisons, regulatory bodies have historically demanded LD_50_ values (the dose lethal to 50% of an animal population) to define the comparative sensitivity of pest and wildlife species. Instead, we developed a replacement assay that used non-lethal dose-response methods to assess the sensitivity of 12 wildlife species and laboratory rats to PAPP that did not require death to be used as an experimental outcome. By establishing the relationship between non-lethal doses of PAPP and the formation of methaemoglobin (MetHb), we found that we could accurately predict doses likely to be lethal. Our estimates very closely approximated existing LD_50_ values determined for PAPP. We argue that laboratory-based lethal-dose bioassays are unsuited to assessing the comparative hazard of toxicants to wildlife species. In contrast, non-lethal assays that use biologically relevant measures can provide much more robust and meaningful indications of relative hazard even in species of high conservation value, where lethal experimentation can rarely be justified.

**Abstract:**

Para-aminopropiophenone (PAPP) is a potent methaemoglobin (MetHb) forming agent used for the lethal control of exotic carnivores and mustelids. To assess the sensitivity of Australian wildlife to PAPP we developed an in vivo assay that did not use death as an endpoint. Sub-lethal dose-response data were modelled to predict PAPP doses required to achieve an endpoint set at 80% MetHb (MetHb_80_). The comparative sensitivity of non-target mammals referenced to this endpoint was found to be highly variable, with southern brown bandicoots (*Isoodon obesulus*) the most sensitive species (MetHb_80_ = 6.3 mg kg^−1^) and bush rats (*Rattus fuscipes*) the most tolerant (MetHb_80_ = 1035 mg kg^−1^). Published LD_50_ estimates were highly correlated with PAPP doses modelled to achieve the MetHb_80_ endpoint (r^2^ = 0.99, *p* < 0.001). Most dose-response data for native mammals were collected in the field or in semi-natural enclosures, permitting PAPP and placebo dosed animals to be fitted with tracking transmitters and transponders and released at their point of capture. A protracted morbidity and mortality was observed only in Australian ravens (*Corvus coronoides*). The combination of sub-lethal dose-response assay and survival data collected in the field provided more relevant information about the actual hazard of pest control agents to non-target wildlife species than laboratory-based lethal-dose bioassays. We discuss the need to replace lethal-dose data with biologically meaningful insights able to define a continuum of toxicological hazards that better serve the needs of conservation and veterinary scientists and wildlife managers.

## 1. Introduction

Since European settlement of Australia, an extensive fauna of exotic and feral species became established throughout the continent [1]. Poison baiting is now used on a landscape-scale to mitigate the impact of introduced pests within agricultural and natural environments [2,3]. Selective lethal agents are sought to mitigate the impacts of introduced predators such as feral cats (*Felis catus*) and red foxes (*Vulpes vulpes*) [4] whilst producing minimal adverse impacts upon non-target native wildlife.

Lethal dose bioassays have remained the principal method used to assess the comparative sensitivity of target and non-target species to prospective pest control agents [5,6,7,8,9,10,11]. The median lethal dose (LD_50_), based upon the method first described by Trevan (1927), provides the principal comparative metric of species sensitivity. However, a requirement for lethal outcomes means that high conservation value species will rarely be used given their genetic value [12] and critical role in species recovery programs [13]. This constrains the scope of toxicological risk assessments and fails to define non-target risk in those species of greatest concern. While variants of the classical lethal bioassay methods can be refined to substantially reduce the scale of animal use, many are not accepted by regulatory agencies [14], frequently due to their poor precision [15]. Accordingly, viable non-lethal assays able to generate data comparable to lethal dose bioassays are needed in order to better define the hazard of prospective pest control agents especially in species with the most precarious conservation status.

In 1981 the Organization for Economic Cooperation and Development (OECD) incorporated the use of the LD_50_ assay in its guidelines for the testing and regulation of chemicals [16], although death as an experimental endpoint was gradually prohibited or restricted in many government jurisdictions [17]. More recently the European Union and OECD guidelines have emphasized the use of alternatives to lethal-dose bioassays [18]. As a consequence, many toxicological risk assessment methods have shifted towards the adoption of predictive models based upon underlying mechanisms of action and biochemical pathways involved in different gradients of exposure [19]. Pathway-based models shown to be *fit for purpose* can be used in tandem with sub-lethal dose-response studies [20] that seek to measure perturbations in relevant physiological or toxicological pathways as alternative endpoints [21]. We sought non-lethal in vivo approaches to assess the sensitivity of a range of Australian wildlife species to para-aminopropiophenone (PAPP), an agent that showed potential as a new predator control agent for wild dogs and exotic foxes.

Orally ingested PAPP is rapidly N-hydroxylated to the active species, para-hydroxylaminopropiophenone (PHAPP) [22] which is a potent methaemoglobin (MetHb) forming agent [23]. Methaemoglobin, arising through the oxidation of haemoglobin in the ferrous valance (HbFe^2+^) to the ferric state (HbFe^3+^), is unable to transport oxygen (O_2_) as oxyhaemoglobin (HbO), or carbon dioxide (CO_2_) as carbaminohaemoglobin. Because O_2_ is required as the final electron acceptor in the electron transport chain to drive aerobic ATP synthase [24], hypoxia arising from severe methaemoglobinaemia has profound consequence for brain function, as the brain is reliant upon aerobic metabolism to maintain sufficient ATP turnover to sustain consciousness and vital cell functions [25]. Lethal-dose bioassays conducted in the early 1940s [26] implied that carnivores and mustelids had a far higher acute sensitivity to PAPP in comparison to most other animal groups [27]. Oral doses of PAPP given to red foxes under laboratory and pen conditions resulted in a toxicosis that appeared far more humane than that achieved using fluoroacetic acid (1080) [28], which remains the principle pest control agent used for carnivore control in Australia [29]. However, as no PAPP toxicity data for Australian wildlife species were available, we developed a sub-lethal assay to establish the relationship between oral PAPP doses and MetHb formation. This permitted the relative sensitivity of a wide range of high conservation value species to be predicted, removing the need to use death as an experimental endpoint.

## 2. Materials and Methods

### 2.1. Selection of Target and Non-Target Populations

The procedures and methods used to obtain, capture and handle wildlife species are described in the supplementary information in more detail (Supplementary). Exotic red foxes and wild dog/dingo hybrids (*Canis lupus familiaris*) were the principal Australian target species and used to validate our alternative experimental endpoints. Non-target mammals and birds present in south-eastern Australia were prioritized if their diets and foraging strategies put them at risk of consuming meat baits used for the routine control of foxes and wild dogs [30]. This included several carnivorous and omnivorous species listed under the *Australian Environment Protection and Biodiversity Conservation Act 1999* including the spotted-tailed quoll (*Dasyurus maculatus*), eastern quoll (*Dasyurus viverrinus*), Tasmanian devil (*Sarcophilus harrisii*) and southern brown-bandicoot (*Isoodon obesulus*). Laboratory rats (*Rattus norvegicus*) were obtained from scientific suppliers. Five other indigenous marsupials, 2 endemic rodents and 2 bird species were also seen as priority species (see Table 1).

### 2.2. Dosage Methods

Prior to PAPP or placebo dosing animals were lightly sedated with 4 mg kg^−1^ intra-muscular (IM) Zoletil (Virbac: Melbourne, Australia). Body weight for animals >5 kg was determined using a digital balance accurate to ±1.0 g while smaller animals were weighed on a digital laboratory balance accurate to ±0.001 g. A stock solution of PAPP dissolved in dimethyl sulfoxide (DMSO) or polyethylene glycol (PEG) permitted maximum PAPP dose concentrations of 375 mg mL^−1^ or 50 mg mL^−1^, respectively. Oral doses were delivered when the anaesthetic was abating and the gag reflex was present. Doses of PAPP (mg kg^−1^) were increased by multiples of 1.5 aiming to produce maximum MetHb concentrations between 70 and 75% in wildlife species. Initially, dogs and foxes were dosed with a wide range of PAPP concentrations using both the DMSO and PEG stock solution in separate trials in order to assess the utility of each formulation and to validate an alternative endpoint for latter trials with wildlife. Dosages using the PEG carrier were achieved by gavage using a dog urine catheter. PAPP + DMSO formulations were delivered to the back of the throat using an ejector system [28]. In small mammals, dosing was achieved by slowly introducing the dose to the back of the tongue using a pipette. Animals were held upright for a further two minutes and their necks massaged to ensure the dose had been swallowed. Formulations of PAPP + DMSO were used exclusively in assessments in non-target wildlife where doses of DMSO alone were used as a placebo.

### 2.3. Blood Sampling and MetHb Determination

Methaemoglobin, as a percentage of total haemoglobin, was determined using a CO-oximeter (OSM 3 Hemoximeter: Radiometer, Copenhagen, Denmark). Quolls, Tasmanian devils, potoroos, bandicoots and pademelons were sequentially blood sampled with a heparinised capillary tube after shaving the hair on the ear to expose a vein that was then swabbed with 70% ethanol before venepuncture with a 23G needle, taking <0.1 mL of blood on each occasion. For larger animals such as foxes and dogs, a dose of 10–15 mg kg^−1^ IM Zoletil was administered, before blood was taken from the cephalic vein or jugular using a 23G needle and heparinised syringe. Blood was collected from small mammals (rats and dunnarts) using the lateral saphenous vein collection method [31]. Birds were sampled from the jugular or brachial vein. In animals dosed with PAPP, blood samples were taken every 20 min until an asymptote of MetHb was detected as the peak MetHb concentration. Thereafter, a sample was taken at 10 min intervals to confirm a decline in MetHb by two sequential readings before the protocol ceased. At the end of the trials, euthanasia of foxes and wild dogs was achieved via an intra-cardiac injection of 10 mL sodium pentobarbital (Lethabarb: Virbac, Sydney, Australia or Mebunat Vet 60: Orion Pharma, Finland) while the animal was under deep anaesthesia induced by an additional IM dose of 15 mg kg^−1^ of Zoletil.

### 2.4. Recovery, Release and Monitoring

While anaesthetized, mammals were implanted with a 1.4 mm × 8 mm RFDI microchip (Trovan ID-100: Microchips Australia, Keysborough, Australia) using the appropriate cannula. Those animals >100 g in body weight were fitted with a single stage 173 MHz transmitter (Supplementary). At the end of the procedure they were placed within a darkened and insulated recovery box or covered cage filled with insulating material for thermal support. Ambulatory animals were provided with water *ad libitum* and observed until fully coordinated and then released at their point of capture. Over the following 4 weeks those exposed to oral doses of PAPP or a placebo dose of DMSO were monitored regularly to determine their survival using a 3-element Yagi antenna and 173 MHz tracking receiver (Titley Electronics: Brendale, Queensland, Australia). Animals that died during the dose-response experiment were excluded from the survival study. Similarly, if animals were euthanised due to injuries sustained during captivity they were also excluded. If the fate of the animal was unknown (if it could not be located) no assumption was made concerning its survival and it too was excluded.

### 2.5. Statistical Analysis

An experimental endpoint (85% MetHb) had been validated after preliminary trials in red foxes [28] and feral cats (Marks, unpublished data) [32], where the Animal Ethics Committee had permitted death as an endpoint in order to validate the utility of non-lethal endpoints using MetHb thresholds. Published fox data for PAPP + DMSO (Marks et al. 2004) were re-analysed in this paper alongside new data developed for PAPP + PEG formulations. To ensure the survival of all wildlife species used, a maximum response of 75% MetHb was sought, from which the alternative endpoint of 80% MetHb (MetHb_80_) was predicted. Peak MetHb concentrations resulting from doses of PAPP were fitted to a statistical model that best described the dose-response relationship with the highest level of significance. Linear and non-linear dose-response models were tested for their strength of fit indicated by R^2^ values, significance and the Akaike Information Criteria [33]. Models were chosen with the assistance of Statistical Package for the Social Sciences (SPSS) (Tablecurve: SPSS Version 14, Chicago, USA). Fitted curves were calculated with 95% confidence and prediction intervals [34] and used to estimate doses of PAPP mg kg^−1^ that would result in 50% (MetHb_50_) and 80% MetHb (MetHb_80_). Validation of the predictive value of the models was sought by regressing the MetHb_50_ and MetHb_80_ predictions against PAPP LD_50_ values for the laboratory rat [35], feral cat (Marks, unpublished data) [36], fox [26], dog [37] and brushtail possum [38]. A Fisher exact test was used to test for significant differences in the survival of all animals in groups that received a PAPP dose or a placebo.

## 3. Results

### 3.1. Dose-Response Models for Red Foxes and Wild Dogs

**Figure 1:**
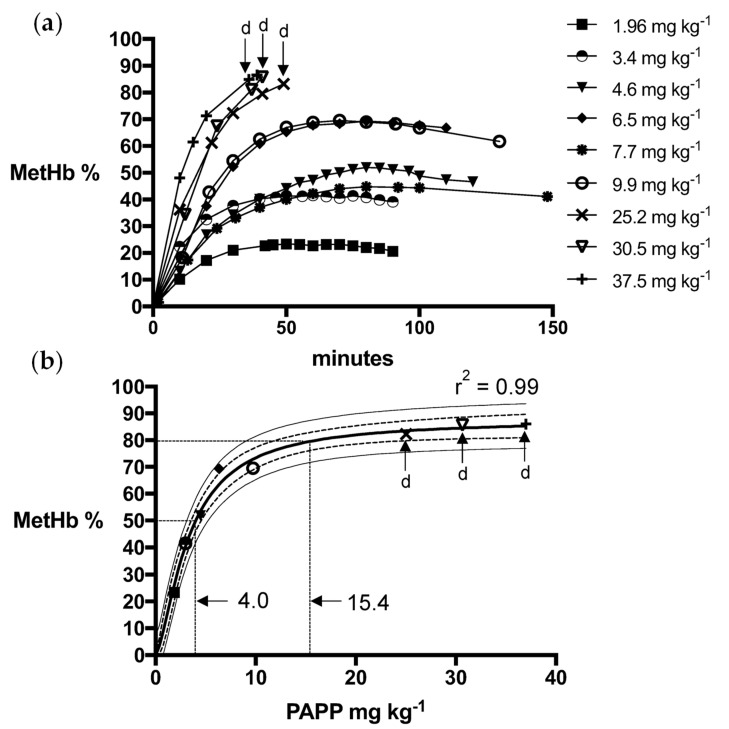
(**a**) Methaemoglobin (MetHb %) response relative to oral dose of PAPP + DMSO in foxes and (**b**) the fitted logistics dose-response model for peak MetHb % with 95% confidence (dashed line) and prediction interval (solid line) and whether death (d) resulted.

**Figure 2:**
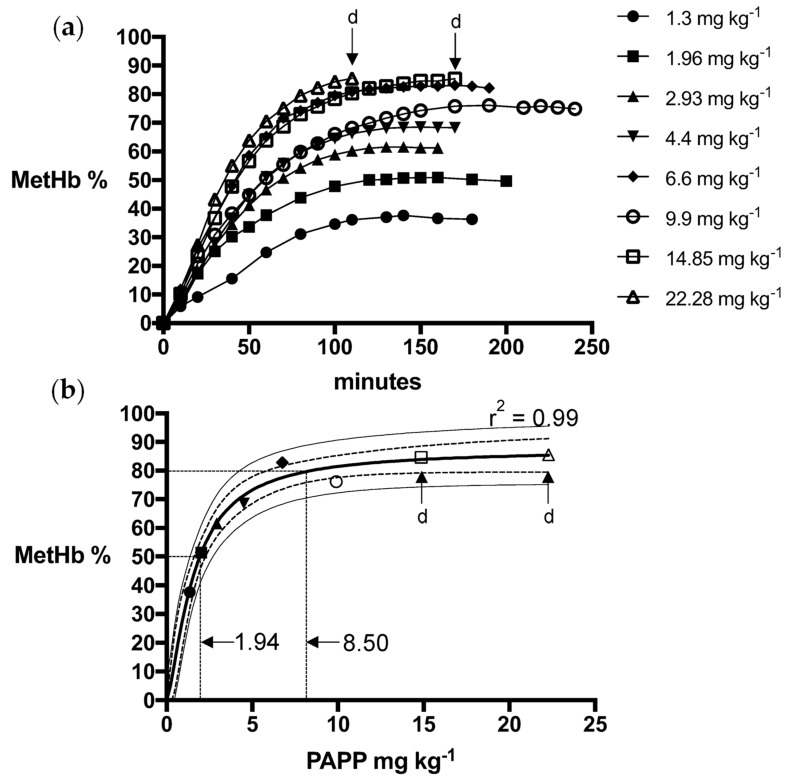
(**a**) Methaemoglobin (MetHb %) response relative to oral dose of PAPP in DMSO in wild dogs and (**b**) the fitted logistics dose-response model for peak MetHb % with 95% confidence (dashed line) and prediction interval (solid line) and whether death (d) resulted.

**Figure 3:**
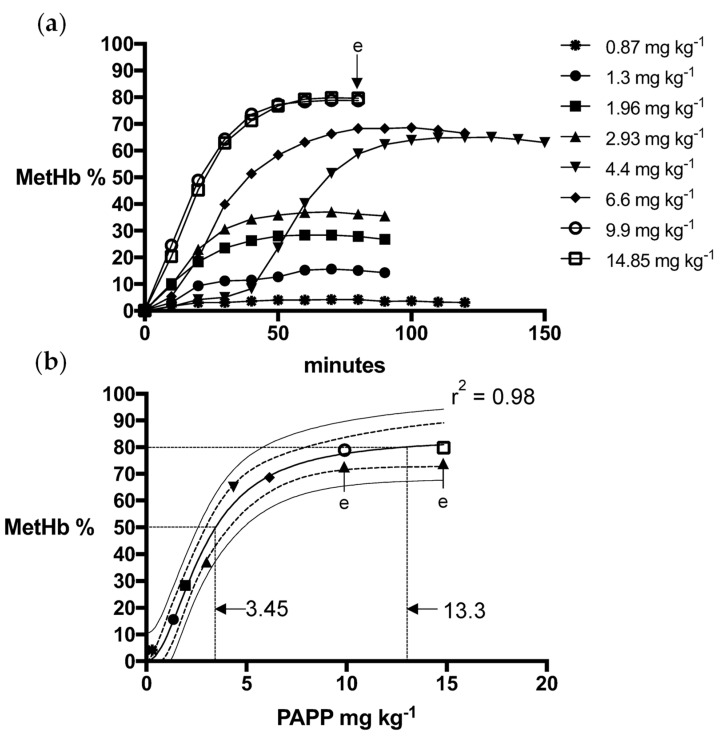
(**a**) Methaemoglobin (MetHb %) response relative to oral dose of PAPP in PEG in foxes and (**b**) the fitted logistics dose-response model for peak MetHb % with 95% confidence (dashed line) and prediction interval (solid line) and when animals were euthanised (e) when they passed the 80% MetHb threshold.

**Figure 4:**
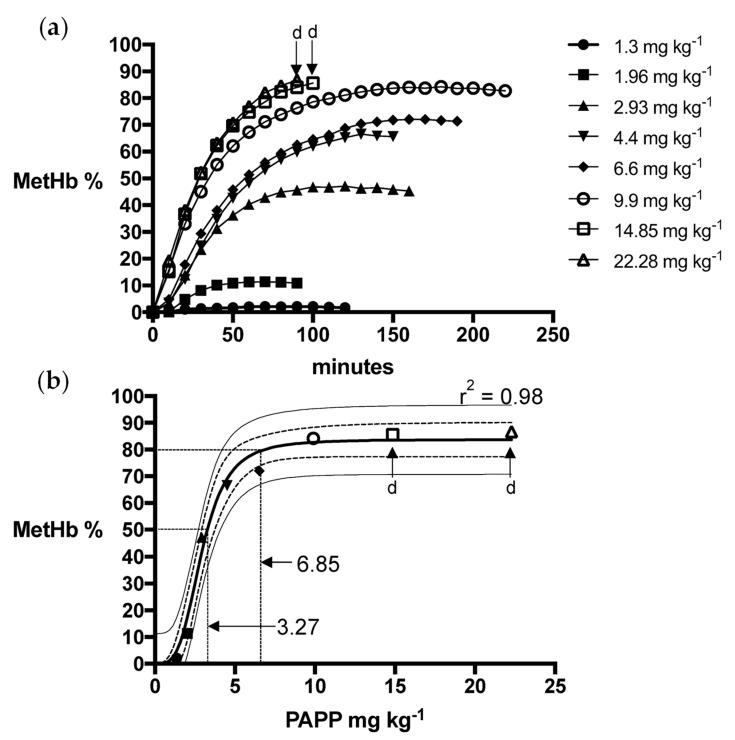
(**a**) Methaemoglobin (MetHb %) response relative to oral dose of PAPP in PEG in wild dogs and (**b**) the fitted logistics dose-response model for peak MetHb % with 95% confidence (dashed line) and prediction interval (solid line) and whether death (d) resulted.

Logistic dose-response models for PAPP + DMSO formulations were a strong fit for fox (R^2^ = 0.99) and wild dog (R^2^ = 0.99) MetHb responses, predicting mean PAPP doses of 15.4 mg kg^−1^ and 8.4 mg kg^−1^, respectively, to attain the MetHb_80_ endpoint (Figure 1 and Figure 2). Using PAPP + PEG formulations, marginally lower mean PAPP doses in foxes (13.3 mg kg^−1^, R^2^ = 0.98) and wild dogs (6.85 mg kg^−1^, R^2^ = 0.98) were predicted to attain the MetHb_80_ endpoint (Figure 3 and Figure 4).

All foxes died if they exceeded the MetHb_80_ threshold in the PAPP + DMSO group (peak MetHb % = 83.2, 85.7 and 86.5). Overall, 4 of 6 wild dogs (peak MetHb = 85.4, 85.5, 85.6 and 86.7%) died in the PAPP + PEG group, with two others vomiting after oral dosing and surviving (peak MetHb = 83.1 and 84.2%), causing data from these individuals to be discarded. Because DMSO + PAPP was not associated with vomiting in foxes or dogs, and was capable of higher PAPP concentrations and low dose volumes, it was used consistently thereafter in all dose-response trials with wildlife species.

### 3.2. Comparative Species Sensitivity to PAPP

Four different models were fitted to non-target data to take account of differences in the dose-response relationship that varied between being highly linear, sigmoidal or exponential in nature. A single fat-tailed dunnart and southern brown-bandicoot received PAPP doses that inadvertently caused MetHb levels to rise to 82% and 82.2%, respectively, resulting in their death. These were the only two unintentional deaths arising from the acute dose-response experiments in wildlife species. The southern brown bandicoot (MetHb_80_ = 6.3 mg kg^−1^) was the only mammal found to be relatively more susceptible to PAPP than wild dogs or foxes. Bush rats (MetHb_80_ = 1035 mg kg^−1^) were the most tolerant mammals, yet large variability in tolerance between rodents was evident, with swamp rats (MetHb_80_ = 26.1 mg kg^−1^) and laboratory rats (MetHb_80_ = 182.7 mg kg^−1^) substantially more sensitive. Similar variation was seen within the dasyurids, with fat-tailed dunnarts (MetHb_80_ = 101 mg kg^−1^), eastern quolls (MetHb_80_ = 277 mg kg^−1^) and Tasmanian devils (MetHb_80_ = 120.3 mg kg^−1^) being comparatively more tolerant compared to spotted-tailed quolls (MetHb_80_ = 27.1 mg kg^−1^). Blood sampling was not attempted in brown antechinus in the field due to their small body mass (<20 g) obviating repeated blood sampling, yet this dasyurid recovered from PAPP doses as high as 571 mg kg^−1^. Both the long-nosed potoroo (MetHb_80_ = 164 mg kg^−1^) and pademelon (MetHb_80_ = 334 mg kg^−1^) responses indicated a much greater tolerance in macropodids compared to foxes and wild dogs. Silver gulls (MetHb_80_ > 1000 mg kg^−1^) appeared to be the most tolerant species overall, as despite a maximum practical dose of 857 mg kg^−1^, the MetHb response did not exceed 7.9%, meaning that no precise estimate of the MetHb_80_ was obtained (Table 1).

Overall, both red foxes and wild dogs were found to be highly sensitive to PAPP relative to most other non-target mammals assessed. Mean doses of PAPP to achieve the MetHb_80_ endpoint in all other species were 34-fold of those required to attain the same endpoint in wild dogs (±21.5-fold, *p* < 0.05) and 20-fold higher for foxes (±11.9-fold, *p* < 0.05). Bush rats required doses 122-fold and 67-fold that of wild dogs and foxes, respectively, to attain the MetHb_80_ threshold. However, southern-brown bandicoots were outliers and susceptible to doses 0.7-fold and 0.4-fold that of wild dogs and foxes (Table 2).

### 3.3. Comparison with Published LD_50_ Data

**Figure 5:**
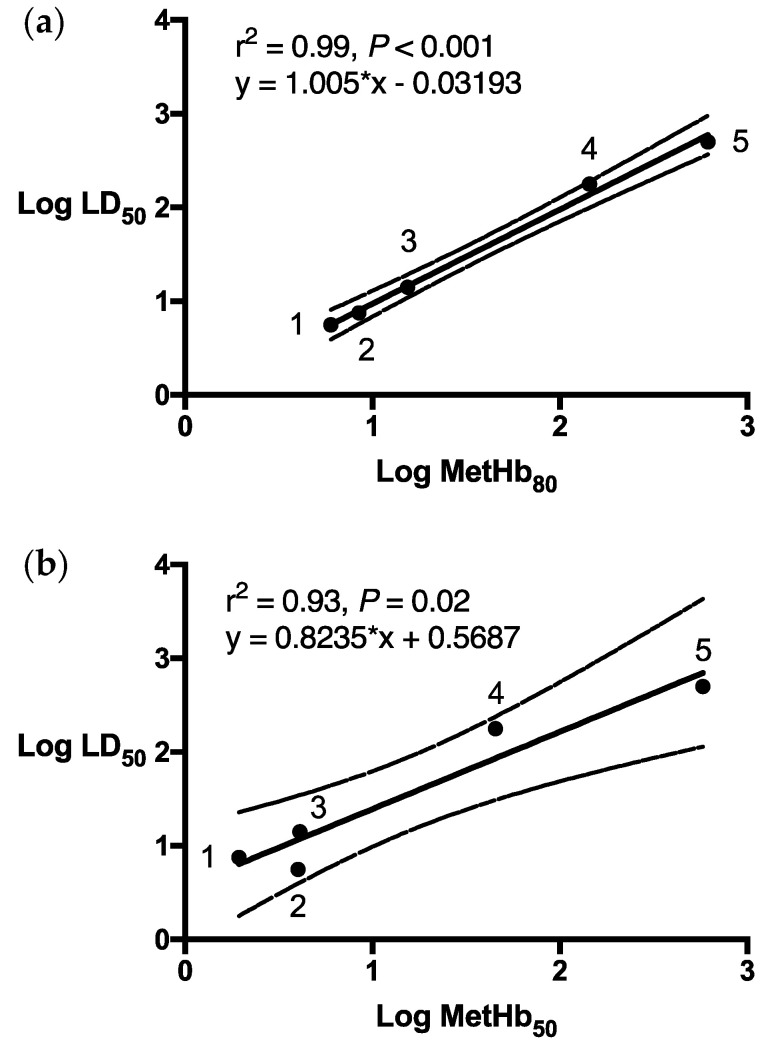
Comparison of log oral PAPP LD_50_ for (1) feral cats, (2) dogs, (3) foxes, (4) laboratory rats and (5) brushtail possums against (**a**) predicted log 80% MetHb level (Log MetHb_80_) and (**b**) log 50% MetHb level (Log MetHb_50_) with 95% confidence.

Log MetHb_80_ estimates based upon dose response data obtained in wild dogs, foxes, feral cats, brushtail possums and brown rats regressed strongly and significantly with log LD_50_ data published for these five species (r^2^ = 0.99, *p* = 0.001). Regression of LD_50_ values with the doses of PAPP modelled to cause a 50% elevation in MetHb (MetHb_50_) produced a weaker correlation (r^2^ = 0.93, *p* = 0.024) with 95% confidence intervals and prediction boundaries indicating a less reliable predictive capacity for the MetHb_50_ estimate (Figure 5a,b).

### 3.4. 10-Day Survival Assessment

Beyond 10 days after the release of instrumented animals the detachment of transmitters and/or their failure due to damage saw a rapid decline in the sample size, making 14-day and 30-day survival monitoring impractical. Radio-tracking and recapture data revealed that 10 days subsequent to release, 77 of 79 PAPP-dosed and 58 of 59 placebo-dosed animals were known to be alive. Overall, survival data for mammals indicated that there was no significant difference in mortality between the groups (*p* = 0.64). However, an Australian raven dosed with 380.7 mg kg^−1^ PAPP died within 24 h after a peak of 67.3% MetHb had been detected in the dose-response trials prior to release. Another raven that received 253.8 mg kg^−1^ (peak MetHb = 68.9%) was unable to fly for 24 h, appearing to recover but died within the 10-day monitoring period. All ravens dosed below 169.2 mg kg^−1^ PAPP survived the 10-day monitoring period. At a dose between 33.42 and 75.2 mg kg^−1^ there appeared to be no noticeable morbidity other than a temporary reduction in physical activity within 2 h after PAPP dosing. Morbidity in ravens appeared to be dose dependent where mild cyanosis was associated with lethargy increasing in severity from 75.2 mg kg^−1^ PAPP (Table 3).

## 4. Discussion

### 4.1. Non-Lethal Data to Model Lethal End-Points

Doses of PAPP predicted to achieve the MetHb_80_ endpoint correlated strongly with known LD_50_ values generated by bioassay. In generating data in 12 non-target wildlife species and the laboratory rat (*n* = 13), 2 of 96 individuals (2.1%) died as a direct result of the dose-response assay procedures where the MetHb_80_ endpoint was unintentionally exceeded. Given an improved understanding of mammalian dose-response relationships after these trials, it is now possible to better anticipate lethal responses and more reliably reduce their occurrence. Nonetheless, the low overall prevalence of mortality in wildlife was highly favourable in comparison to that hypothetically required by three OECD-approved LD_50_ methods modified to reduce animal use. Here, between 2 and 40 animals would have been used in each comparison [14] that for 13 species would require between 26 and 520 individuals in order to rank the toxicity of PAPP into six different categories based upon 10-fold dose increments [39]. At best, such a categorical classification provides an extremely low resolution of comparative hazard for wildlife species, where in the case of PAPP susceptibility most of the species we assessed would be grouped into only two categories of sensitivity.

Death marks the very end of a continuum of pathophysiological changes caused by a gradient of toxicant doses. Lethal dose bioassays measure only dichotomous outcomes (death or survival) and not a spectrum of prepathological and pathological consequences leading to death [40]. In contrast, pathway-based studies can be used to define thresholds of severity or adverse effects [41,42] revealed by changes in continuous or discrete responses related to dose. MetHb-forming compounds, where an increasing gradient of adverse effects are directly related to dose, are well suited to pathway-based assessments.

In vertebrates, MetHb is normally present at low concentrations (<1%), often due to autoxidation of haemoglobin in the presence of O_2_ [43]. Exposure to MetHb-forming compounds [44] can rapidly increase the concentration of MetHb in blood, potentially resulting in a dose-dependent hypoxia and inhibition of aerobic ATP production [45] that cause a continuum of signs and symptoms associated with a worsening hypoxia. In humans, 10% MetHb caused by PAPP administration produced measurable performance deficits under dynamic loads [46], although concentrations <20% MetHb may otherwise be asymptomatic. Beyond 30% MetHb, symptoms such as dizziness, fatigue, headache and weakness [47] are frequently reported by patients and are well known indicators of advancing hypoxia. Cardiac arrhythmias and other signs of pathological hypoxia become evident at >55% MetHb [48]. Peak MetHb values >70% are generally associated with unconsciousness and carry a risk of death if sustained [49]. Studies in populations of red foxes showed that MetHb fractions >80% were required before brain death resulted, while below this threshold all foxes appeared to recover uneventfully (Marks et al. 2004) as was the case with wild dogs and mammals in our current study. Concentrations of MetHb >87% were reported to be reliably lethal in dogs [26,50], implying that the MetHb_80_ endpoint lies below doses that may correspond with an LD_99_ estimate. Adopting a lower endpoint (MetHb_80_) was appropriate for our comparative studies to assure that death was avoided in wildlife species, in part because some data suggested that deaths in smaller mammals may occur marginally closer to the MetHb_80_ threshold (at 82% MetHb in the only two cases that resulted in a lethal endpoint). Hence, we attempted to constrain doses of PAPP in wildlife species to maximum responses ranging between 70 and 75% MetHb that appeared to be well tolerated, ideally permitting the projection of the MetHb_80_ value over a narrow range.

### 4.2. Assessing Survival in the Field

Australian ravens were the only species observed to experience a protracted morbidity and delayed mortality. Although the aetiology was unqualified during this trial, more recent insights into the impact of PAPP on avifauna was obtained in later studies that better elucidated the nature of the protracted toxicosis in some birds [51].

Our protocol of releasing and remotely monitoring wildlife survival differed markedly from conventional lethal-dose studies that are routinely conducted in controlled laboratory environments. From its first description, the LD_50_ value was known to be dependent upon the experimental conditions under which it was derived [52], where variations in factors such as temperature, food and thermal support, that were known to influence mortality or survival, could be controlled [53]. However, the maintenance of captive wildlife species in controlled environments does not adequately reflect the heterogeneity of stressors normally encounter within their habitats. No wildlife species have uniformly available resources, as is often the case in the laboratory with the provision of food, water and thermal support. Periods of negative energy balance are frequently encountered by wildlife [54] that must also defend their body temperature by behavioural as well as autonomic responses [55]. Therefore, captive trials cannot mimic the stressors and environmental conditions encountered by wildlife after ingesting bait poisons in situ. Instead, captivity introduces novel stressors that elicit a large array of hormonal and physiological changes that may be highly species-specific [56], resulting in acute and chronic stress affecting the health and welfare of species quite differently [57].

It has been observed that the captive animal is physiologically quite distinct from its wild counterpart. Captivity alone may confound a wide range of experimental results, making toxicity data taken from captive wildlife populations potentially unreliable indicators of outcomes in free-ranging species [58]. This led us to collect dose-response data in the field so that captured animals could be assessed using sub-lethal dose-response assays and released as soon as practical, their longer-term survival monitored remotely under environment conditions normally encountered within their own habitats.

### 4.3. Re-Thinking How the Hazard of Bait Toxicants Is Assessed in Non-Target Wildlife

Since first described in 1927 [52], lethal-dose bioassays became a cornerstone of efforts to achieve standardization in drug and chemical risk evaluation [59]. Regulatory bodies used LD_50_ assay data from laboratory animals to routinely classify the relative toxicity and hazard of substances [14] as a proxy for the human lethal dose data [60]. While the use of laboratory animals to model human sensitivity to toxicants met with variable success [61], lethal dose bioassays were not specifically developed as methods to assess the comparative hazard of toxicants to wildlife species or to predict their impact upon animal health and biological fitness. In contrast, wildlife managers, conservation scientists and veterinary clinicians are concerned with qualifying and quantifying the impact of poisons upon the fitness and conservation status of individuals and populations. Already well-known limitations in the interpretation of lethal-dose bioassay data become far more problematic when they are used to investigate the comparative hazard of toxicants within complex communities of wild vertebrates. Genotypic polymorphism is correlated with variation in enzyme systems involved with the activation and biotransformation of xenobiotic compounds [62,63] where the presence of intraspecific and interspecific variation in toxicokinetics and toxicodynamics is unassailable [64]. Unlike selectively bred laboratory species [65] that are typically used in lethal-dose bioassays, wildlife species are far more polymorphic, requiring toxicology assay methods that are fit for purpose and able to routinely survey the relative hazard of poisons to large numbers of wildlife species, enabling intra-specific variations in sensitivity to be revealed. For example, the co-evolution of wildlife species with fluoroacetic acid bearing plants in the west of Australia resulted in a large comparative difference in regional tolerance to 1080 compared to conspecifics in the east of the country where such plants are absent [66]. Moreover, the selection of greater resistance in rabbit (*Oryctolagus cuniculus*) populations frequently exposed to 1080 was revealed by bioassays [67]. Each is an example of when ongoing monitoring for changing sensitivity in both target and non-target species is needed in order to determine the overall cost-benefit of poison baiting. Although currently unexplored, similar forms of heterogeneity in sensitivity to PAPP can be investigated using non-lethal dose-response assays and survival studies conducted in the field.

Non-lethal and pathway-based assays that measure appropriately selected continuous or discrete variables can also denote dose-dependent adverse effects thresholds, such as the no observable effect level (NOEL) and the lowest observed adverse effect level [LOAEL] [41,42]. The NOEL denotes doses that have no measurable impact on health and welfare, while the LOAEL is associated with the lowest dose associated with the onset of adverse effects. Each were employed to better define the relative hazard of PAPP to New Zealand bird species, generated by a non-lethal dose-response assay [51]. Historically, the emphasis on the statistical significance of death and survival as markers of the dose-response relationship [68], is a legacy of a regulatory culture primarily concerned with the classification of human risk, not one seeking to determine the comparative risk of toxicants to wildlife populations. Determining only the probability of death from exposure is an extremely course measure of hazard to wildlife species. Instead, methods most suited to describing the hazard of bait toxicants to a wide range of wildlife species should seek to establish thresholds of acceptable and unacceptable animal health and welfare.

## 5. Conclusions

Australian mammals were found to vary greatly in their comparative sensitivity to PAPP (MetHb_80_ = 6.3–1035 mg kg^−1^). Modelling dose-dependent MetHb responses permitted MetHb_80_ endpoints to be predicted, that were strongly correlated with known LD_50_ values for five species. Survival data were collected in the field, avoiding the need for captivity in a laboratory setting where animals would be subjected to a range of novel stressors. Survival data collected over a 10-day period allowed protracted effects upon Australian ravens to be identified. Overall, sub-lethal dose-response methods were a viable alternative to lethal-dose bioassays for establishing the comparative hazard of PAPP to Australian wildlife.

Lethal dose bioassays measure only dichotomous data—death and survival. In contrast, assays that monitor continuous variables linked to dose-dependent pathophysiological changes may be correlated with changing animal welfare states. Non-lethal assays potentially allow thresholds to be set to demarcate acceptable and unacceptable welfare impacts associated with perturbations in pathophysiological markers at levels well below those that cause mortality. Non-lethal assays ensure that sensitivity data can be collected in a wide range of species, including high conservation value species, where lethal experimentation can rarely be justified.

## Figures and Tables

**Table 1:** Oral PAPP dose in mg kg^−1^ formulated with DMSO estimated to produce 50% (MetHb_50_) and 80% (MetHb_80_) MetHb based upon fitted models with upper and lower prediction estimates (*p* < 0.05).

Common Name	Specific Name	Model *	*n*	m	r^2^	*F*	*p*	MetHb_50_		MetHb_80_	
								Dose	*p* < 0.05	Dose	*p* < 0.05
								mg kg^−1^	mg kg^−1^	mg kg^−1^	mg kg^−1^
Australian raven	*Corvus coronoides*	1	7	1	0.97	220	<0.0001	33.82	35.1, 33.2	-	-
Brushtail possum	*Trichosurus vulpecula*	2	9	0	0.94	101	<0.0001	574.8	604, 590.1	614.9	760.0, 604.0
Bush rat	*Rattus fuscipes*	3	7	0	0.99	433	<0.0001	293	205.5, 144.7	1035	1217.0, 634.0
Eastern quoll	*Dasyurus viverrinus*	3	9	0	0.91	110	<0.0001	78.3	170.5, 25.8	277	504.5, 107.7
Fat-tailed dunnart	*Sminthopsis crassicaudata*	3	6	1	0.99	299	<0.0001	40	61.9, 22.7	101	140.5, 67.9
Laboratory rat	*Rattus norvegicus*	3	8	0	0.97	234	<0.0001	46.5	76.5, 25.5	182.7	257.0, 106.0
Long-nosed potoroo	*Potorous tridactylus*	2	6	0	0.95	85.1	<0.001	87.0	121.8, 54.0	164.3	201.9, 127.4
Red fox	*Vulpes vulpes*	4	9	3	0.99	371	<0.0001	4.0	5.3, 3.2	15.4	38.0, 11.48
Silver gull	*Chroicocephalus novaehollandiae*	4	7	0	0.97	67	<0.001	>1000	-	>1000	-
Brown-bandicoot	*Isoodon obesulus*	3	5	1	1	397	<0.001	2.96	3.3, 2.6	6.3	6.9, 5.8
Spotted-tailed quoll	*Dasyurus maculatus*	2	9	0	0.97	346	<0.0001	12.9	18.9, 9.7	27.1	29.3, 20.1
Swamp rat	*Rattus lutreolus*	2	8	0	0.96	190	<0.0001	14.5	18.8, 10.4	26.1	29.9, 22.4
Tasmanian devil	*Sarcophilus harrisii*	4	8	0	0.99	960	<0.0001	27.2	32.4, 22.9	120.3	156.0, 71.5
Tasmanian pademelon	*Thylogale billardierii*	3	7	0	0.91	80	<0.0003	88.5	191.0, 127.9	334	396.0, 103.0
Wild dog	*Canis lupus familiaris*	4	9	2	0.99	268	<0.0001	1.94	2.7, 1.5	8.5	19.8, 4.4
Total			114								

* (1) $\mathrm{In}y=a+be^{-x}$, $\left(2\right)\;\mathrm{In}y=a+bx^{3}$, (3) $y=ax^{b},\;$(4) $y=a+\frac{b-a}{1+\left(\frac{c}{x}\right)}$.

**Table 2:** Comparative species sensitivity referenced to the multiple of red fox and wild dog sensitivity based on the dose of PAPP mg kg^−1^ required to cause 80% MetHb in each with upper and lower confidence estimates (*p* < 0.05).

Common Name	× Dog	*p* < 0.05	× Fox	*p* < 0.05
Australian raven	17.4 *	18.1, 17.1 *	8.5	8.8, 8.3
Brushtail possum	72.3	89.4, 71.1	39.9	49.4, 39.2	
Bush rat	121.8	143.2, 74.6	67.2	79.0, 41.2	
Eastern quoll	32.6	59.4, 12.7	18.0	32.8, 7.0	
Fat-tailed dunnart	11.9	16.5, 8.0	6.6	9.1, 4.4	
Laboratory rat	21.5	30.2, 12.5	11.9	16.7, 6.9	
Long-nosed potoroo	19.3	23.8, 15.0	10.7	13.1, 8.3	
Red fox	1.8	4.5, 1.4	-	-	
Silver gull	>118	-	>64.9	-	
Southern brown-bandicoot	0.7	0.8, 0.7	0.4	0.4, 0.4	
Spotted-tailed quoll	3.2	3.4, 2.4	1.8	1.9, 1.3	
Swamp rat	3.1	3.5, 2.6	1.7	1.9, 1.5	
Tasmanian devil	14.2	18.4, 8.4	7.8	10.1, 4.6	
Tasmanian pademelon	39.3	46.6, 12.1	21.7	25.7, 6.7	
Wild dog	-	-	0.6	1.3, 0.3	
Mean	34.1		20.1		
*p* < 0.05	21.5		11.9		

* Based upon MetHb_50_ comparison.

**Table 3:** Summary of fate of animals known to be alive (KTBA) for a 10-day period after release as determined from recapture, direct observation within captive colony or radio-tracking data for all animals dosed with PAPP dissolved in DSMO (PAPP) or those given a placebo consisting of DMSO alone (placebo).

Species	PAPP			Placebo		
	Monitored	KTBA	Died	Monitored	KTBA	Died
Antechinus	5	5	0	-	-	-
Brushtail possum	10	10	0	6	6	0
Eastern quoll	9	9	0	9	9	0
Little Australian raven	6	4	2	6	6	0
Long-nosed potoroo	6	6	0	6	6	0
Tasmanian pademelon	8	8	0	8	8	0
Silver gull	5	5	0	7	6	1
Southern brown bandicoot	4	4	0	6	6	0
Spotted-tailed quoll	5	5	0	1	1	0
Swamp rat	10	10	0	5	5	0
Tasmanian devil	9	9	0	5	5	0
Total	77	75	2	59	58	1

## Data Availability

Data is available for fair use to support further scientific research upon request to the corresponding author.

## Supplementary Materials

The following are available online at https://www.mdpi.com/article/10.3390/ani13030472/s1.
