# Supplementary material for: Non-Lethal Dose-Response Models Replace Lethal Bioassays for Predicting the Hazard of Para-Aminopropiophenone to Australian Wildlife
Source: Animals (Basel). 2023 Jan 29;13(3):472. doi: 10.3390/ani13030472 (PMC9913665; doi:10.3390/ani13030472)
Supplement: Supplementary file 1 [file animals-13-00472-s001.zip › animals-2093851-supplementary.pdf]

Supplementary

Trapping methods

Wild dogs were captured using standard techniques using soft-catch traps [61] and held at the Robert Wicks Research Station (Inglewood, Queensland). Foxes were also trapped using soft-catch devices and general trapping methods that have been previously described [62]. Laboratory rats and dunnarts were purchased from the Monash University central animal house. Bush rats were taken from French Island in bush blocks adjacent to roadways. Aluminium Elliot Traps were baited with a mixture of rolled oats, honey and peanut butter and set in a well-drained and sheltered site protected with vegetation cover from the wind and morning sun. Small mammals taken from the traps were released into a canvas bag and transferred to a rodent box containing cover (shredded newspaper), food (dry dog food) and a water *ad libitum*. Antechinus, rats and bandicoots were trapped with wire cage traps (550 x 200 x 200 mm) or Elliot traps (230 x 100 x 100 mm) set one hour before sunset and baited with a lure of apple, rolled oats, sugar and other fruits. Traps were set in a well drained and sheltered sites protected from the wind and morning sun. Pademelons were captured at a free-ranging colony at the Mt Pleasant Laboratories (Launceston) and potoroos were obtained at a colony held at LaTrobe University by hand netting. Quoll trapping procedures followed those used by DPIWE (Tasmania) and approved by a Tasmanian AEC or a University of Sydney AEC. Quolls were captured in wire cage traps (60 x 60 x 30 cm) or small PVC tube traps. Traps were set in areas of natural vegetation at least one hour before sunset and baited with chicken meat. Trap inspection occurred at least every 12 hours and as regularly as the site permitted. Capture procedures for Tasmanian devils followed those used by DPIWE (Tasmania) and approved by a Tasmanian AEC. Capture used large PVC tube traps (approximately 1 m x 0.3m) baited with chicken meat or commercial dried dog food. To avoid population disruption and concerns over the spread of DFTD, captured devils were held in quarantine facilities for the two-week period. At each site these consisted of pens measuring approximately 15 x 15 x 1.8 m. Little Australian raven and silver gull were trapped in large cage traps (60 x 60 x 30 cm) set during the day and baited with chicken meat and bread or by using a custom made net gun. All brushtail possums were trapped and used *in situ* at the Fitzroy Gardens (Melbourne, Victoria). Upon capture animals were housed individually within 550 x 200 x 200 mm cages. Possums were provided with food and water *ad libitum* after recovery from anaesthesia. They were captured using standard cage traps with a treadle plate release or baited trigger arm using fresh apple and were set directly below known possum feeding trees. Inspections were conducted every two hours and the cages of trapped animals were covered with a sack. All procedures were conducted within a 24-hour period (ideally during the same evening) and possums were released at the point of capture.

Radio tracking

A 1cm^2 area of fur was shaved to stubble and a single-stage, glue-on transmitter attached to the skin and hair surface with Vetbond (Therapon Veterinary Suppliers: Melbourne, Australia). A single stage 173 MHz

transmitter (Titley Electronics: Brendale, Queensland, Australia) was attached to a shaved portion of skin at the end of the tail and secured with surrounding hair with Vetbond adhesive that was bound with Micropore tape (3M: Therapon Veterinary Suppliers: Melbourne, Australia) and impregnated with latex adhesive. In bandicoots, quolls and pademelons, transmitters were attached to the base of the tail and supported by a ring of Elastoplast tape that was permeated with adhesive. Antechinus were too small to facilitate transmitter attachment. In little Australia raven and silver gulls small < 3 g transmitters were glued in place, following a method that has been used in much smaller birds (starlings) without reported welfare affects. Transmitters were attached to the underside of the two central rectrices, as close as possible to the base of the feathers (about 8 mm from the skin). Ethanol was used to remove oil and dirt from the feather shafts and to provide better adhesion. Strings on the front of the transmitter were attached to the two central rectrices with a double surgeon's knot. A layer of 5-min epoxy was applied to the feather shafts and the underside of the surgical tubing. The tubing was pushed against the feather shafts, forming a glue line between the feather shafts and the tubing. A single piece of string was then wrapped around both central rectrices and the transmitter was tied off to hold the transmitter securely against the feather shafts while the epoxy set. Once the installation was complete a layer of talcum powder was dusted over the fresh epoxy to make sure that it did not stick to adjacent feathers. Instrumented animals were located regularly by radio telemetry using a 3 element Yagi antenna and scanning receiver (Titley Electronics: Brendale, Queensland, Australia) and at the end of a two week period after initial trapping, recovery was attempted.
